# From limbs to leaves: common themes in evolutionary diversification of organ form

**DOI:** 10.3389/fgene.2015.00284

**Published:** 2015-09-08

**Authors:** Remco A. Mentink, Miltos Tsiantis

**Affiliations:** Department of Comparative Development and Genetics, Max Planck Institute for Plant Breeding Research, Cologne, Germany

**Keywords:** evolution and development, leaflet formation, digit formation, patterning versus post-patterning, morphological diversity

## Abstract

An open problem in biology is to derive general principles that capture how morphogenesis evolved to generate diverse forms in different organisms. Here we discuss recent work investigating the morphogenetic basis for digit loss in vertebrate limbs and variation in form of marginal outgrowths of angiosperm (flowering plant) leaves. Two pathways underlie digit loss in vertebrate limbs. First, alterations to digit patterning arise through modification of expression of the *Patched 1* receptor, which senses the *Sonic Hedgehog* morphogen and limits its mobility in the limb bud. Second, evolutionary changes to the degree of programmed cell death between digits influence their development after their initiation. Similarly, evolutionary modification of leaf margin outgrowths occurs via two broad pathways. First, species-specific transcription factor expression modulates outgrowth patterning dependent on regulated transport of the hormone auxin. Second, species-specific expression of the newly discovered REDUCED COMPLEXITY homeodomain transcription factor influences growth between individual outgrowths after their initiation. These findings demonstrate that in both plants and animals tinkering with either patterning or post-patterning processes can cause morphological change. They also highlight the considerable flexibility of morphological evolution and indicate that it may be possible to derive broad principles that capture how morphogenesis evolved across complex eukaryotes.

A key question in biology is how morphological diversity is generated. Although plants and animals evolved multicellularity independently, within each kingdom conserved gene regulatory networks (hereafter termed networks) control the development of one or more body parts. In this context evolution operates as a “tinkerer,” being strongly influenced by the materials currently at hand as well as prior history (Jacob, 1977; Davidson and Erwin, 2006; Pajoro et al., 2014; Sorrells et al., 2015). Consequently, considerable constraints exist on the evolution of new traits (Pires-daSilva and Sommer, 2003; Davidson and Erwin, 2006; Carroll, 2008; Pires and Dolan, 2012) raising the question of how evolutionary changes to networks that control development may circumvent these constraints.

Both theoretical arguments and empirical evidence suggest that regulatory sequence variation has greater potential for the generation of morphological change than coding sequence variation (Stern, 2000; Carroll, 2008). This is likely because regulatory sequences tend to be organized in highly modular *cis* elements, leading to their mutation having a lower propensity to generate pleiotropic effects that would compromise development (Stern, 2000; Carroll, 2008; Rebeiz et al., 2015). However, to what degree this broad principle manifests itself in different evolutionary lineages and how precisely the balance of conservation versus divergence of different networks creates morphological diversity remain open questions (Stern and Orgogozo, 2008, 2009; Rebeiz et al., 2015).

Other than knowing the types of genetic changes underlying the generation of morphological diversity, an understanding of evolution requires determining how, when and where those genetic changes influence morphogenesis. For example, it remains largely unclear if particular stages or aspects of development tend to be preferentially amenable to evolutionary tinkering. Does evolution primarily target developmental processes that are active during early stages of organ development; or are such early stages less favored by evolution, owing to the potential risk of causing pleiotropic effects that will influence later stages of development? Does diversity largely arise through tinkering with later acting developmental programs that fine-tune organ form after the more fundamental patterns have been laid down? Studies on the emergence of novel insect pigmentation patterns in closely related species suggest that later developmental stages (e.g., the insect pupal stage) might be more readily available for evolutionary tinkering (Wittkopp and Beldade, 2009). However, definitive answers to these questions are likely still to come and will depend on the particularities of the system that is under investigation, including its evolutionary history, its modularity, the type of trait being studied and its degree of integration with other traits. Nevertheless, one way to approach these problems in a unified fashion when comparing diverse organisms is to consider whether and how evolution influences patterning and post-patterning modes of development. Patterning processes act to impart positional information, for example through the use of morphogen concentration gradients, and facilitate correct distribution of cellular identities within tissues (Kondo and Miura, 2010; Rogers and Schier, 2011). Post-patterning processes, on the other hand, serve to sculpt emerging tissues and organs typically after their identity has previously been determined. For example, post-patterning processes may operate by removing superfluous cells through apoptosis or by adjusting the growth rates of specific populations of cells within the organ (Coen et al., 2004; Suzanne and Steller, 2013). Notably, this distinction between “post-patterning” and patterning does not exclude the possibility that patterning genes may have persistent effects in developmental time, including post-patterning stages (Salazar-Ciudad et al., 2003; McGregor et al., 2007; Werner et al., 2010).

Two recent papers have explored the significance of patterning versus post-patterning events on development by studying digit loss in mammals and leaf shape formation in angiosperms and revealed a strong link between altered, species-specific gene expression domains and morphological variation. Both studies suggest considerable versatility in how evolutionary tinkering with developmental processes can ultimately arrive at similar phenotypes.

Cooper et al. (2014) studied the evolutionary changes that resulted in convergent digit loss in different mammalian species. A mammalian limb (such as a leg) is attached to the body at one (proximal) end and has 1 to 5 anteroposteriorly distinct digits (e.g., toes) at the other (distal) end. Limbs develop from the limb bud through the sequential action of several distinct signaling centers (Figure 1A; Butterfield et al., 2010). Bone morphogenetic proteins (BMPs) specify the formation of the apical ectodermal ridge (AER) at the distal end of the limb bud, from which fibroblast growth factors (FGFs) are secreted to stimulate proximodistal outgrowth (Lewandoski et al., 2000; Pizette et al., 2001; Boulet et al., 2004). The morphogen Sonic hedgehog (SHH) is secreted from the posterior limb bud to direct both digit patterning and expansion of the hand- or footplate to accommodate all digits (Harfe et al., 2004; Towers et al., 2008). Subsequent digit elongation is controlled by FGFs secreted from the AER and in later stages BMPs sculpt the limb by inducing apoptotic cell death within interdigital tissue in concert with the transcription factor *Msx2* (Marazzi et al., 1997; Ferrari et al., 1998; Sanz-Ezquerro and Tickle, 2003).

**FIGURE 1:**
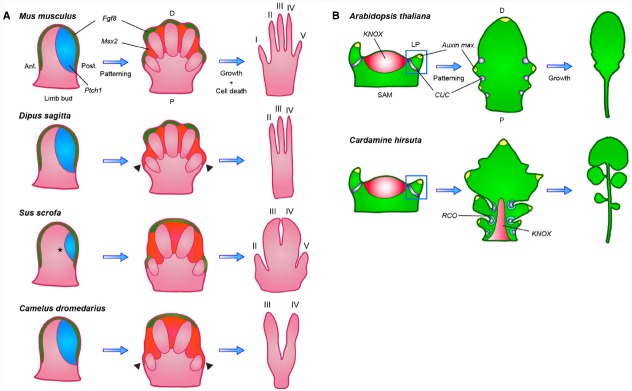
**Diversification of both patterning and post-patterning processes contributed to morphological variation of leaves and limbs. (A)** In the vertebrate limb bud *Fgf8 (green)* expression from the apical ectodermal ridge (AER) stimulates proximodistal outgrowth, while posteriorly expressed *Ptch1 (blue)*, through sequestration, creates a posterior to anterior *SHH* gradient that patterns the individual digits. In later stages, *Fgf8* is maintained only in the AER, overlying digits that will completely form, while *Msx2 (orange)* expression is turned on in the interdigital tissue, triggering apoptosis. In the 3-toed jerboa and camel, expanded *Msx2* expression causes the regression of the outermost digits (indicated by arrowheads). In the pig, *Ptch1* expression is reduced to eliminate digit I (indicated by a star), a change that is not observed in the closely related camel. Ant, anterior; Post, posterior; P, proximal; D, distal. **(B)** Simple and dissected leaves similarly initiate as small leaf primordia (LP) from the shoot apical meristem (SAM) at sites where auxin maxima *(yellow)* are defined by *CUC (purple)* and *PIN1* expression, but only dissected leaves reactivate *KNOX (red)* expression to suppress leaf cell differentiation. This allows the initial marginal outgrowths, patterned by *CUC* and *PIN1*, to develop into leaflets instead of serrations. Species-specific local expression of *RCO (blue-green)* in *Cardamine hirsuta* restricts cellular growth within leaf sinuses, thereby allowing separation of individual leaflets through modulation of local growth rates. P, proximal; D, distal.

Cooper et al. (2014) studied the possible relevance of these pathways to digit loss in 3- and 5-toed jerboas (small, desert-dwelling rodents that develop a varying number of digits on the hind limb between different species) and mice, as well as in ungulates (hoofed animals with 1 to 4 toes). In jerboas, they observed no differences in patterning gene expression but rather found expanded domains of apoptotic cells in 3-toed jerboa hind limbs, surrounding tissue otherwise destined to form digits I and V. Specifically, they found that expression of *Msx2* was expanded in the 3-toed jerboa hind limb, likely causing increased cell death (Figure 1A). They obtained comparable results with 1-toed horse embryos, where *Msx2* expansion correlated with removal of digits II and IV. This shows a convergent evolutionary event in which an apoptotic pathway normally used to remove interdigital tissue was co-opted by regulatory changes to act in truncating digit outgrowth.

By expanding their study to even-toed ungulate species, Cooper et al. (2014) found a striking flexibility in modes of digit loss. In pigs the expression of *Patched 1 (Ptch1)*, a *Shh* receptor, is reduced toward the posterior limb bud. *Ptch1* acts to restrict the spread of *Shh* by sequestration, thus reduction in *Ptch1* expression leads to an expanded region of *Shh* activity and more uniform expression of its target genes, presumably causing a shift in limb axis symmetry to the space between digits III and IV (Figure 1A; Chen and Struhl, 1996; Butterfield et al., 2009). These findings were corroborated by a second group that showed a similar reduction of *Ptch1* expression in cow limb buds (Lopez-Rios et al., 2014). These authors also demonstrated that *cis* regulatory divergence of *Ptch1* renders it unresponsive to *Shh* signaling in a negative feedback loop. Remarkably, when Cooper et al. (2014) examined embryos of camel, a third ungulate, they observed no modification of *Ptch1* expression, but instead an expansion of apoptosis and *Msx2* expression, resembling the case in 3-toed jerboas and horses (Figure 1A; Cooper et al., 2014). These results indicate that in species of the same taxonomic order, such as camels and pigs (both members of the Artiodactyla or even-toed ungulates), fundamentally different mechanisms can be modified to achieve similar organ modifications, revealing considerable flexibility in evolutionary pathways. A conclusion that is additionally in line with the fact that Cooper et al. (2014) did not recover any evidence for evolutionary tinkering with the *HoxD* regulatory landscape, which has previously been identified and hypothesized to be a good candidate for vertebrate digit diversification, owing to its highly modular nature (Montavon et al., 2011). These findings raise the question of how broadly this flexibility in evolutionary tinkering with either growth or patterning occurred during evolution of complex eukaryotes.

A recent paper by Vlad et al. (2014) establishes that a comparable logic helps explain diversification of leaf shapes in plants of the Brassicaceae family. Brassicaceae, like other flowering plants, form either simple leaves, consisting of an entire blade with smooth, serrated or lobed margins, or dissected leaves, comprising individual leaflets. Both types of leaves develop from leaf primordia that initiate from the pluripotent shoot apical meristem (SAM). *KNOTTED-LIKE HOMEOBOX (KNOX)* transcription factors are expressed in the meristem to maintain its organ-generating potential (Hay and Tsiantis, 2006; Barkoulas et al., 2007). Transport of the plant hormone auxin through the *PIN-FORMED 1* (*PIN1*) efflux transporter, coupled to a self-reinforcing feedback of auxin on PIN1 expression and polarization, likely creates sequential local auxin activity maxima at the flanks of the SAM. This process appears to be self-organizing and the resulting auxin maxima are required for sequential primordium development (Reinhardt et al., 2003; Heisler et al., 2005; Jonsson et al., 2006; Smith et al., 2006). *CUP-SHAPED COTYLEDON (CUC)* genes mark the leaf primordium boundary and allow its separation by repressing growth at the flanks (Aida et al., 1997; Hibara et al., 2006). *CUC*s and *PIN1* also function together to pattern the leaf margin, as *CUCs* likely repress growth at the boundaries of serrations or leaflets, while *PIN1* generates auxin maxima at the sites of their outgrowth. Notably, in this context CUCs likely both repress growth at the flanks of marginal outgrowths and promote their outgrowth at least in part via promoting generation of an auxin maximum at their tip (Nikovics et al., 2006; Barkoulas et al., 2008; Blein et al., 2008; Koenig et al., 2009; Kawamura et al., 2010; Ben-Gera et al., 2012). In the *Arabidopsis thaliana* leaf margin, *CUC2* directs PIN1 localization to form local auxin maxima while auxin feeds back to repress *CUC2*, creating the repeated pattern of leaf serrations along the leaf margin (Bilsborough et al., 2011). *KNOX* genes, then, are expressed in dissected leaves and differentiate these from simple leaves by retarding cellular differentiation, thus rendering the leaf competent to form leaflets in response to *PIN1* dependent auxin maxima (Hay and Tsiantis, 2006; Barkoulas et al., 2008; Kimura et al., 2008; Bar and Ori, 2014). Similarly *CUC1*, a redundantly acting paralogue of *CUC2*, is expressed in the dissected leaves of *Cardamine hirsuta* but is confined to the leaf meristem boundary in its simple-leaved relative *A. thaliana.* These observations indicate that evolutionary tinkering with auxin-based patterning mechanisms through alterations in expression of upstream transcription factors such as *KNOX* and *CUC* may be a major route for generating diversity in leaf shapes (Figure 1B; Barkoulas et al., 2008; Blein et al., 2008; Piazza et al., 2010; Hasson et al., 2011; Finet and Jaillais, 2012; Bar and Ori, 2014).

Until recently, no genes had been identified that specifically influence leaflet formation without also affecting meristem function or leaf initiation. Such findings suggested that leaflets form through the redeployment of processes that acted earlier in development during leaf initiation (Bar and Ori, 2014; Vlad et al., 2014). To identify novel regulators of leaf complexity, Vlad et al. (2014) conducted a forward genetic screen for genes required for leaflet formation in *C. hirsuta*. They identified the *REDUCED COMPLEXITY (RCO)* homeobox gene, of which a loss of function allele simplifies the leaf without causing pleiotropic phenotypes, suggesting a specific requirement for *RCO* in leaflet formation. *RCO* evolved in the Brassicaceae family from a gene duplication of *LATE MERISTEM IDENTITY 1* (*LMI1*); originally identified in *A. thaliana* as a floral regulator (Saddic et al., 2006). They found that *RCO* is specifically expressed at the base of leaflets (Figure 1B), while *LMI1* is expressed more distally, in a complementary pattern, along the leaf margins. *RCO* does not appear to influence PIN1-mediated auxin patterning, but instead functions by repressing cellular growth between individual leaflets in *C. hirsuta*, a post-patterning process that allows leaflet separation. *RCO* was lost in *A. thaliana* during evolution, contributing to its leaf simplification, but re-introducing *RCO* into *A. thaliana* drives expression in basal regions of the leaf and increases leaf complexity, partially reversing the consequences of evolution. These results, together with a follow-up study in the sister species *Capsella rubella* and *Capsella grandiflora* by Sicard et al. (2014), suggest that *RCO* is a key regulator of leaf shape and diversity in the Brassicaceae and provide a striking example of organ shape diversification by tinkering with local growth regulation at the flanks of a growing organ primordium (Vlad et al., 2014). Another notable aspect of the *RCO* study is that this gene was discovered through performing a forward genetics study in *C. hirsuta* and could not have been found in *A. thaliana*, where the gene has been lost, thus highlighting the importance of unbiased studies in diverse taxa for understanding the genetic basis for the evolution of form.

Taken together, these two studies illustrate how evolution can exploit both patterning and post-patterning processes to create morphological diversity in both plants and animals. It will be interesting to explore whether bias might exist for variations of either kind or for particular developmental pathways across different kingdoms. For example, plants and animals have evolved distinct biophysical properties and morphogenetic strategies that pose different constraints for evolution. Whereas animal morphogenesis involves the use of large-scale apoptosis and cell migration, these mechanisms are used to more limited extent (Gunawardena, 2008; Fendrych et al., 2014) or not at all respectively in plants. This is because rigid cell walls in plants somewhat complicate the use of both these mechanisms during development: cell walls typically remain after apoptosis, thereby constraining developmental options, while they make cell migration impossible by preventing the sliding of cells alongside each other. These fundamental differences in the cellular underpinnings of development suggest that morphological diversity in plants mostly arises through tinkering with regional growth rates and growth directionality (Coen et al., 2004), consistent with the findings of Vlad et al. (2014) These particularities of plants, however, do not preclude that changes to such growth-related processes can also contribute to the evolution of animal form (Abzhanov et al., 2004; Wu et al., 2004). In any event, independent of the organism studied, morphology is determined by processes that take place at different levels of organization and yield the final form through complex feedback loops of genetic regulation, signaling and tissue growth (Salazar-Ciudad and Jernvall, 2010; Kennaway et al., 2011; Prusinkiewicz and Runions, 2012). Conceptualizing how activity of gene regulatory networks creates organ shape is consequently not solely intuitive. The computer science and developmental biology interface offers a promising path for resolving such problems in a predictive fashion (Lewis, 2008; Green et al., 2010; Prusinkiewicz and Runions, 2012; Sheth et al., 2012). Quantitative investigations of morphogenesis and the genetic basis of its variation in different organismal lineages will allow us to build a general picture of how organ diversity is generated and maintained. Such studies should also help us understand the basis for and limits of predictability of morphological evolution.

## Conflict of Interest Statement

The authors declare that the research was conducted in the absence of any commercial or financial relationships that could be construed as a potential conflict of interest.
